# Synergistic combination of a topologically invariant imaging signature and a biomarker for the accurate prediction of symptomatic radiation pneumonitis before stereotactic ablative radiotherapy for lung cancer: A retrospective analysis

**DOI:** 10.1371/journal.pone.0263292

**Published:** 2022-01-31

**Authors:** Kenta Ninomiya, Hidetaka Arimura, Tadamasa Yoshitake, Taka-aki Hirose, Yoshiyuki Shioyama

**Affiliations:** 1 Division of Medical Quantum Science, Department of Health Sciences, Graduate School of Medical Sciences, Kyushu University, Higashi-ku, Fukuoka, Japan; 2 Faculty of Medical Sciences, Division of Medical Quantum Science, Department of Health Sciences, Kyushu University, Higashi-ku, Fukuoka, Japan; 3 Department of Clinical Radiology, Graduate School of Medical Sciences, Kyushu University, Higashi-ku, Fukuoka, Japan; 4 Division of Radiology, Department of Medical Technology, Kyushu University Hospital, Higashi-ku, Fukuoka, Japan; 5 Ion Beam Therapy Center, SAGA HIMAT Foundation, Tosu, Saga, Japan; University of Engineering and Technology Taxila Pakistan, PAKISTAN

## Abstract

**Objectives:**

We aimed to explore the synergistic combination of a topologically invariant Betti number (BN)-based signature and a biomarker for the accurate prediction of symptomatic (grade ≥2) radiation-induced pneumonitis (RP+) before stereotactic ablative radiotherapy (SABR) for lung cancer.

**Methods:**

A total of 272 SABR cases with early-stage non-small cell lung cancer were chosen for this study. The occurrence of RP+ was predicted using a support vector machine (SVM) model trained with the combined features of the BN-based signature extracted from planning computed tomography (pCT) images and a pretreatment biomarker, serum Krebs von den Lungen-6 (BN+KL-6 model). In all, 242 (20 RP+ and 222 RP–(grade 1)) and 30 cases (8 RP+ and 22 RP–) were used for training and testing the model, respectively. The BN-based features were extracted from BN maps that characterize topologically invariant heterogeneous traits of potential RP+ lung regions on pCT images by applying histogram- and texture-based feature calculations to the maps. The SVM models were built to predict RP+ patients with a BN signature that was constructed based on the least absolute shrinkage and selection operator logistic regression model. The evaluation of the prediction models was performed based on the area under the receiver operating characteristic curves (AUCs) and accuracy in the test. The performance of the BN+KL-6 model was compared to the performance based on the BN, conventional original pCT, and wavelet decomposition (WD) models.

**Results:**

The test AUCs obtained for the BN+KL-6, BN, pCT, and WD models were 0.825, 0.807, 0.642, and 0.545, respectively. The accuracies of the BN+KL-6, BN, pCT, and WD models were found to be 0.724, 0.708, 0.591, and 0.534, respectively.

**Conclusion:**

This study demonstrated the comprehensive performance of the BN+KL-6 model for the prediction of potential RP+ patients before SABR for lung cancer.

## 1. Introduction

Stereotactic ablative radiotherapy (SABR) is a non-invasive treatment for early-stage non-small cell lung cancer (NSCLC). Previous studies have shown that the overall survival and progression-free survival of patients treated with SABR are comparable to those of lobectomy, although it is a much less invasive procedure [1, 2]. However, SABR can cause symptomatic (grade ≥ 2) radiation-induced pneumonitis (RP+) in 28% of patients [3, 4]. Although most cases of RP are manageable, a few cases are severe, and there is a risk of mortality [3]. This raises the demand for predicting the occurrence of RP+ before SABR to support radiation oncologists in decision-making for radiotherapy.

An antigen Krebs von den Lungen-6 (KL-6), mucin-like glycoprotein, has been recognized as a biomarker of pulmonary epithelial cell injury [5–8]. Besides it is widely known for its association with the activity of interstitial pneumonia (IP), a number of studies reported the usefulness of the KL-6 for the prediction of RP+ prior to SABR [5–7, 9]. They mentioned that patients with elevated KL-6 levels showed the tendency to have RP+ after SABR. Therefore, prescreening of KL-6 is suggested to decide the strategy for SABR in lung cancer patients [5–7]. However, since KL-6 is associated with RP+ as well as the existence of lung cancer itself [10, 11], the predictive performance of pretreatment KL-6 may not be sufficient (S1 Table).

The potential of planning computed tomography (pCT) image signatures in the prediction of RP status after SABR has been explored in previous radiomics studies [12, 13]. Hirose et al. utilized histogram and texture features extracted from pCT and wavelet decomposition (WD) images, which have been widely used in radiomics studies, to construct the image signature and prediction model using the least absolute shrinkage and selection operator logistic regression (LASSO-LR) [13]. Moran et al. attempted to develop a LR model for the classification of the RP status based on the image features extracted from the pCT as well as the post-SABR diagnostic CT images [12]. Although their model demonstrated the feasibility for RP prediction, their area under the receiver operating characteristics (ROC) curve (AUC) could not reach a value higher than 0.76 [12, 13]. The prediction performance is not sufficient for clinical settings. Further, to our knowledge, none of these studies have investigated the impact of the combination of image signature and KL-6 in RP prediction.

Pre-existing pulmonary diseases (prePDs) have been recognized as predictive factors for the occurrence of RP after radiation treatments, including SABR [14–18]. The prePDs associated with RP can be categorized into interstitial pneumonia (IP) and chronic obstructive pulmonary disease (COPD) [14]. It has been reported that patients with the IP had a higher risk of RP+ [15, 16], whereas patients with COPD had a lower risk of RP+ [14, 17, 18]. Importantly, IP and COPD cause structural changes of lung regions, which have been observed in pathological images [19–21]. Consequently, those microstructural changes of lung regions also appear in CT images. IP increases X-ray attenuation as a result of dense fibrotic remodeling of the lung structure [19, 20, 22]. The remodeling results in ground-glass opacities that derive spatially heterogeneous pixel value distributions on CT images. In contrast, the COPD molds lower-attenuation areas, which can be observed as cavitation on the CT images, and are caused by a lack of alveolar surrounding airways [21, 23]. These prePDs could cause pulmonary structural alteration and lead to heterogeneous and/or cavitated lung textures caused by low and/or high attenuation areas on CT images.

We have developed a novel topological image processing technique utilizing Betti numbers (BNs), which represent the number of connected components (b0) and holes (b1), to preserve the intrinsic heterogeneous patterns and cavitation of objects. BN-based image features have demonstrated feasibility in the quantification of tumor heterogeneity and cavitation for prognostic prediction [24] or identification of epidermal growth factor receptor mutations among patients with NSCLC [25]. Further, it has shown robustness against variability in imaging parameters and patient populations [24, 25]. Therefore, we hypothesized that RP pulmonary structural patterns with prePDs on pCT images can be characterized using the versatile topological image processing technique based on BNs. Consequently, the BN signature could be a promising predictive factor for RP before SABR.

Thus, we aimed to develop a comprehensive image signature based on BNs to accurately predict the occurrence of RP+ using pCT images before SABR for lung cancer. Furthermore, we investigated its synergistic relationship with KL-6 to consider the practical use of the BN-based signature in combination with this biomarker. This is the first study exploring the feasibility of a combination of the robust image features based on the BN and the biomarker KL-6. The accurate prediction of the RP+ could potentially guide physicians to determine the optimal dose prescription to avoid severe RP+ in personalized SABR.

## 2. Materials and methods

### 2.1. Clinical cases

A total of 272 patients who underwent SABR for NSCLC were retrospectively enrolled in this study. Patient data for the training dataset were randomly selected during the period between August 2003 and July 2013. As for the test dataset, we collected all the available cases with the RP+, and randomly selected the grade 1 RP (RP–) cases in the period between April 2014 and March 2018, so that the number of cases in both groups was not too different from each other. By doing so, we assumed that the bias to the majority class can be avoided in the evaluation of the performance of the proposed models. [26–29]. When the number of cases in either major or minor class is too small (or high), the model would have a high pseudo accuracy by predicting all the cases as belonging to the major class; but when it comes to the minor class, the performance deteriorates [26–29]. Therefore, the number of RP–was reduced to avoid inappropriate evaluation, which lead to the significant differences in the ratio of RP+ and RP–between the training and test datasets.

The KL-6 testing is a part of the standard protocols in our hospital. Therefore, the KL-6 levels are tested for as many patients as possible. The pCT images for the training dataset were acquired between August 2003 and July 2013 using a CT scanner (Mx 8000, Philips Healthcare, Amsterdam, The Netherlands) with the following scanning parameters: tube voltage, 120 kV; in-plane pixel size, 0.98 mm; and slice thickness, 2.0 mm. Conversely, the pCT images for the test dataset were scanned between April 2014 and March 2018 using a different scanner (Aquilion Prime, Canon Medical Systems, Otawara, Japan) with the same scanning parameters. The patient setups for the image acquisition were performed in the standardized protocol in the Kyushu University Hospital. RP grades were scored using the Common Terminology Criteria for Adverse Events version 4.0 (CTCAE v.4.0), based on clinical assessment and imaging. Anisotropic pCT images and RT structures including lung regions and gross tumor volumes (GTV) were transformed into isotropic images with an isovoxel size of 0.98 mm, using linear and shape-based interpolations, respectively.

This retrospective study was performed with the ethical approval of the institutional review board of our hospital and written informed consent was waived because of the retrospective design. All of the methods were carried out in accordance with the Declaration of Helsinki.

### 2.2 Development of the BN-based signature

#### 2.2.1 Topological image processing and feature extraction

**Fig 1:**
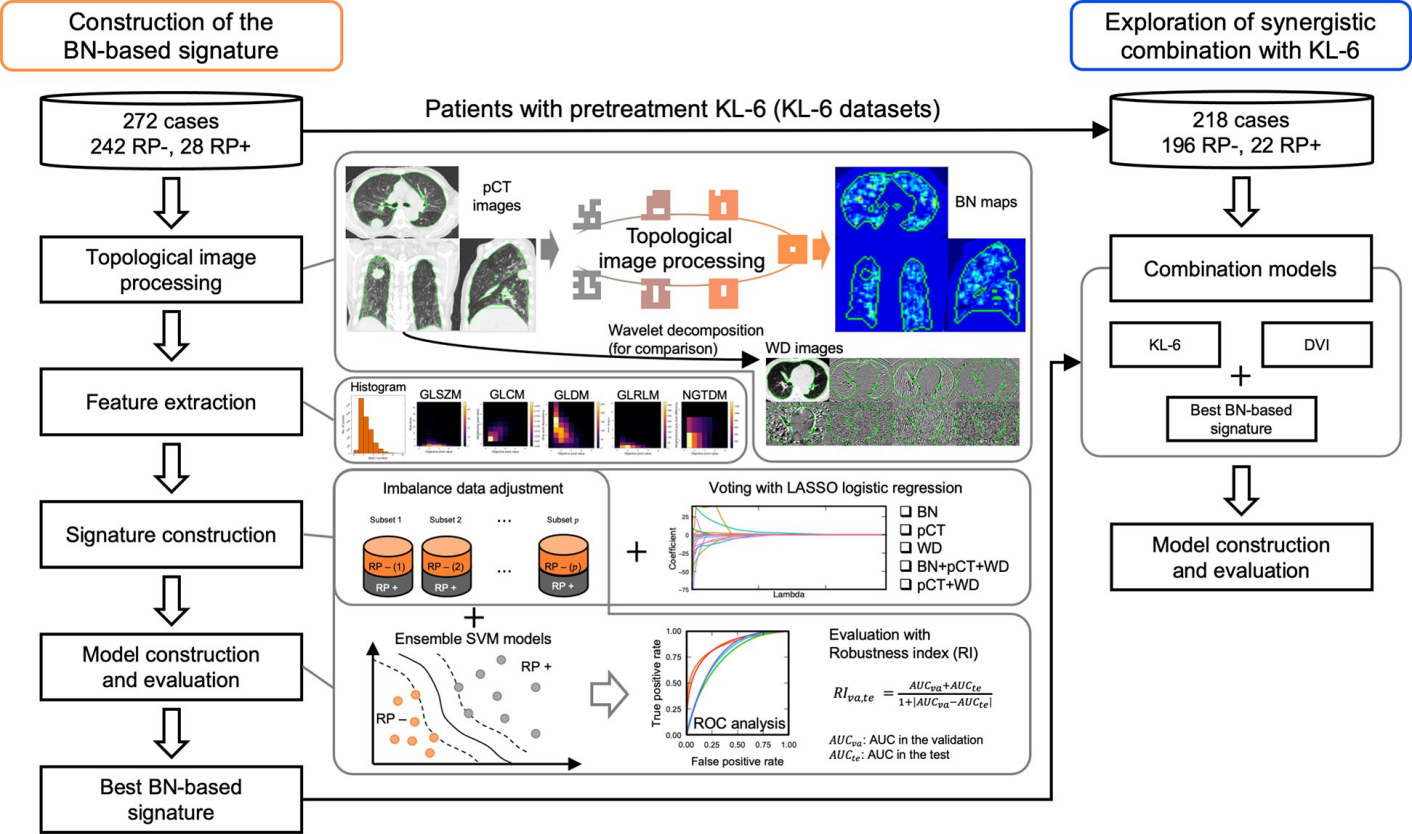
The overall workflow of the present study designed to achieve two major objectives: Construction of the BN-based signature and exploration of synergistic combination with known predictive factors. This study consisted of two main sections. First, the most feasible set of image features (signature) was determined by evaluating their performance in the prediction of RP+. Then, the combination of the image signature and well-known predictive factors including KL-6 and DVI was explored for further improvement of the predictive performance of the model. pCT: planning computed tomography, BN: Betti number, WD: wavelet decomposition, GLSZM: gray-level size zone matrix, GLCM: gray-level co-occurrence matrix, GLDM, gray-level dependence matrix, GLRLM: gray-level run-length matrix, NGTDM: neighborhood gray-tone difference matrix, RP: radiation-induced pneumonitis. LASSO: least absolute shrinkage and selection operator, SVM: support vector machine, KL-6: Krebs von den Lungen-6, DVI: dose volume indices.

Fig 1 illustrates the overall workflow of this study. First, the most feasible set of image features (signature) was determined by evaluating their performance in the prediction of RP+. Then, the combination of the image signature and well-known predictive factors including KL-6 and dose volume indices (DVI) was explored for further improvement of the predictive performance of the model. BN-based image features were extracted from the lung regions on the axial, coronal, and sagittal planes of the pCT images, which included the centroid of the GTV. In all, 47,616 BN-based features were obtained from 512 BN maps (256 b0 maps and 256 b1 maps) that characterize topologically invariant heterogeneous characteristics of lung regions on the pCT images by applying 93 feature calculations (18 histogram-based features and 75 texture-based features, S2 Table). The integrated histogram and texture matrices were obtained from the BN maps for the three-plane pCT images to compute the image features. The computation of the BN maps was performed by counting b0 and b1 in local regions of 256 binary images through a convolutional computation procedure using four kernel sizes (5, 7, 9, and 11 pixels) and five shifting pixels (1, 2, 3, 4, and 5 pixels) [24, 25]. To obtain the 8-bit pCT images, the original CT value range of -1350 to 150 Hounsfield units was converted to 8 bits (0 to 255). The binary images were derived from these 8-bit pCT images by thresholding, with values ranging from 0 to 255.

To compare the feasibility of the BN features with the conventional ones, 93 pCT-based features and 744 WD-based features were extracted from the original and wavelet-decomposed pCT images, respectively, using three-dimensional calculation algorithms for the same 93 features. Wavelet decomposition was performed using the high (H)-pass filter and low (L)-pass filter based on the coiflet 1 mother wavelet. Eight WD images were obtained by applying either H or L filters in the x, y, and z directions [LLL, LLH, LHL, LHH, HLL, HLH, HHL, and HHH (each of characters either L or H represents a low- or high-pass filter applied to the x, y, or z direction)].

The extraction of image features was performed using PyRadiomics package version 3.0.1 in Python 3.8.

#### 2.2.2 Signature construction

**Fig 2:**
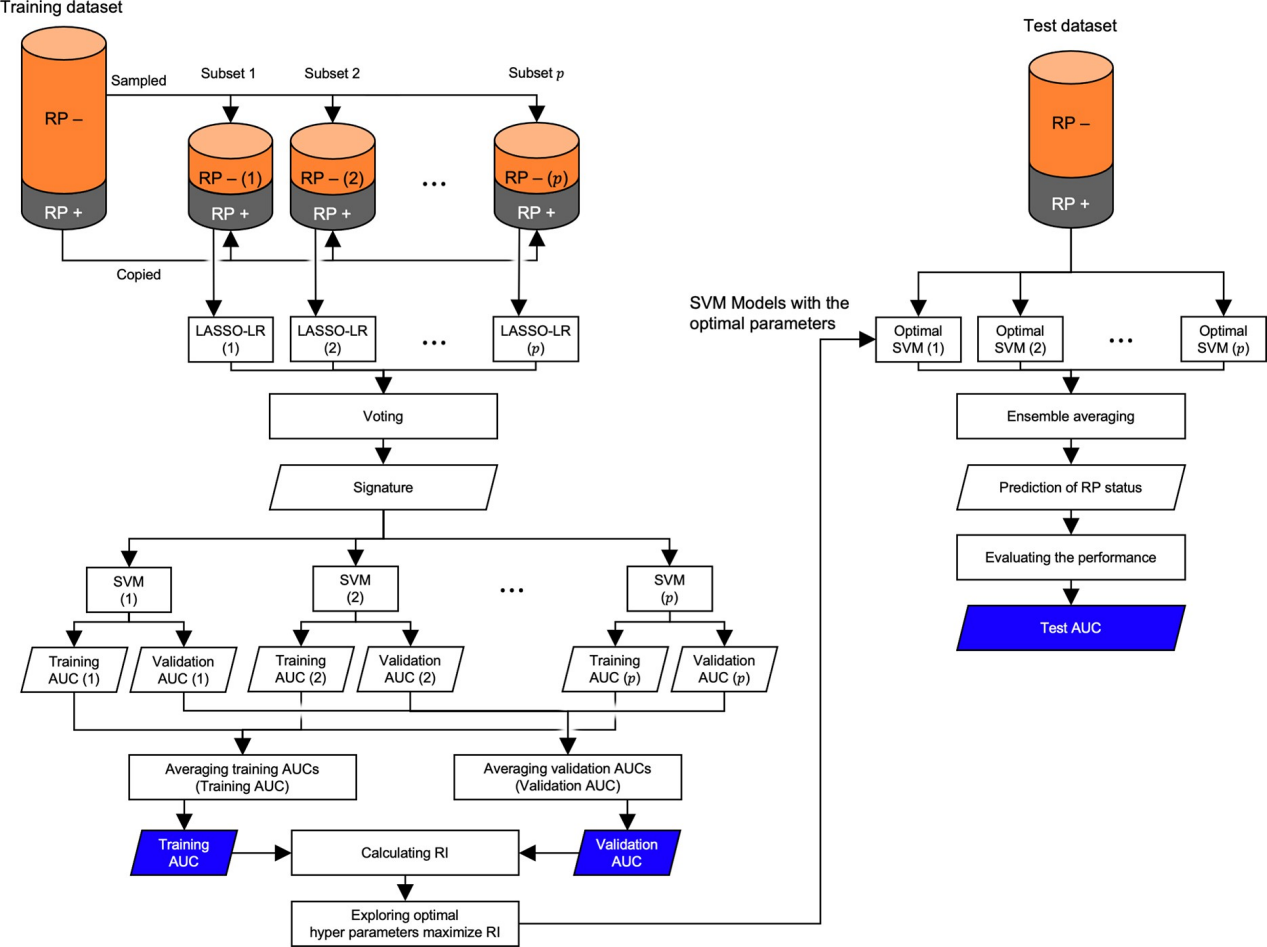
Scheme of the imbalance data adjustment of patients with symptomatic (grade ≥2) radiation pneumonitis positive (RP+) and negative (RP−) for a voting-based feature selection and an ensemble support vector machine (SVM) model construction. LASSO-LR: least absolute shrinkage and selection operator logistic regression; SVM: support vector machine; AUC: area under the receiver operating characteristics curve; RI: robustness index.

The signatures were constructed using frequency-based voting with the LASSO-LR model. Fig 2 shows the procedure for frequency-based voting based on an imbalance adjustment strategy proposed by Schiller et al. [30]. The most frequent features were included in the signature. When features showed the same frequency (ties), those with a higher sum of absolute coefficients in the LASSO-LR models were selected. These frequencies were obtained from the non-zero coefficients of several LASSO-LR models constructed with subsets of the training dataset. The LASSO-LR model for each subset was optimized by maximizing the partial log likelihood while minimizing the L1 norm of the coefficients for the RP prediction in a leave-one-out cross-validation [31]. The subsets were constructed so that the numbers of RP+ and RP− cases were the same or similar. The RP+ cases were copied, and the RP− cases were randomly sampled to each subset. The number of subsets, *p* was determined using the following equation:

$$p=\left\lfloor \frac{N_{-}}{N_{+}}\right\rfloor ,$$ (1)

where *N*_−_ and *N*_+_ indicate the number of RP–and RP+ cases, respectively. ⌊⋅⌋ represents floor function. We used 11 subsets, as *N*_−_ and *N*_+_ were found to be 222 and 20, respectively (41 cases for two subsets and 40 cases for nine subsets). This strategy not only balances the impact of both groups in the construction of the LASSO-LR model but also constructs a consensus signature as a result of voting.

We also constructed a signature with a combination of the BN, pCT, and WD features (BN+pCT+WD model) to investigate the impact of the conventional image features in the RP+ prediction model. The feasibility of the conventional combination consisting of pCT and WD features (pCT+WD model) was also investigated because they were reported to perform better in combination rather than a single implementation of either pCT or WD features [13].

#### 2.2.3 Model construction and evaluation

The occurrence of RP+ was predicted using support vector machine (SVM) models based on the signature. To balance the difference between the number of RP+ and RP− cases, an imbalance adjustment algorithm was also applied to train the SVM models with the same subsets used in the signature construction (Fig 2). Optimal hyperparameters of the SVM were explored with respect to kernels (linear, Gaussian, and sigmoid), soft margin parameters C (ranging from 0.1 to 10), and gamma values (ranging from 0.1 to 10). Parameter optimization was performed based on the robustness index (RI), which defined higher total and lower differences in the AUCs between the training and validation. The RI between the training and validation is defined as follows:

$${RI}_{tr,va}=\frac{{AUC}_{tr}+{AUC}_{va}}{1+|{AUC}_{tr}-{AUC}_{va}|},$$ (2)

where *AUC*_*tr*_ and *AUC*_*va*_ are the AUCs for the training and validation, respectively. In this study, the training and validation AUCs were obtained by averaging the AUCs from the subsets (indicated by blue rhombi in Fig 2). The SVM model in each subset was validated using the leave-one-out cross-validation.

The feasibility of the models was compared with respect to the AUCs, RI, accuracy, sensitivity, and specificity based on the prediction made by ensemble averaging the outputs from reconstructed SVMs in the subsets. The reconstructed SVMs were obtained by training the SVM model for all cases in each subset using the optimal hyperparameters. The averaged SVM output was used to calculate the AUC for the test. The number of features in the signature was explored from 1 to 10 to maximize the RI for validation and testing. RI was calculated from the AUCs for validation and testing. The accuracy, sensitivity, and specificity of the models in the test were calculated based on the ROC curves with the optimal threshold value determined by the minimization of the Euclidean distance between the ROC curve and the point representing a true positive rate of one and a false positive rate of zero.

### 2.3 Exploration of synergistic combination with KL-6

To assess the impact of KL-6 in combination with the BN signature, the SVM model was constructed using the combined features of the BN signature and KL-6 (BN+KL-6) based on the KL-6 dataset using the same SVM construction algorithm described in Section 2.2.3.

Since the feasibility of the DVI of lung volume receiving doses greater than 5, 10, and 20 Gy (V5, V10, and V20) and mean lung dose (MLD) has been reported in previous studies [32, 33], we also constructed an SVM model with BN and DVI (BN+DVI).

### 2.4 Statistical analyses

In the demographic distributions of the patients (Tables 1 and 2), the Mann–Whitney U tests were used to assess significant differences in terms of continuous quantities. In contrast, chi-squared tests were applied for the other categorical variables. Statistically significant differences and 95% confidence intervals (CIs) among the AUCs in the validation and test were assessed using the bootstrap method with 2000 iterations (significance threshold; p<0.05). To compute the statistics regarding the AUCs, we used ensemble outputs from the reconstructed SVMs by feeding the signatures for the training and test datasets. All analyses were performed using R-4.0.3 (http://www.r-project.org/).

**Table 1:** Demographic distributions of the patients and significant differences between the training and test datasets.

	Training dataset	Test dataset	p-value (testing method)
Total number of cases	242	30	
RP grade			
Grade = 1	222	22	4.952×10^−3^ (Chi-squared test)
Grade ≥ 2	20	8	
Pre-treatment KL-6 availability			
Available	193	25	0.823 (Chi-squared test)
Unavailable	49	5	
Pre-treatment KL-6 quantity (U/mL, min-max (median))	115–1853 (262)	134–678 (283)	0.745 (Mann-Whitney U-test)
Age (y, min-max (median))	51–92 (77)	54–90 (74)	0.722 (Mann-Whitney U-test)
Sex			0.511 (Chi-squared test)
Male	150	21	
Female	92	9	
Stage			0.146 (Chi-squared test)
	185	27	
T2N0M0 (stage IB)	57	3	
Dose and fraction			
12 Gy × 4 Fr. (48 Gy)	236	25	4.00 × 10^−3^ (Chi-squared test)
13 Gy × 4 Fr. (52 Gy)	2	3	
6 Gy × 10 Fr. (60 Gy)	4	2	
Dose prescription method			
Isocenter	142	10	1.200 × 10^−2^ (Chi-squared test)
D95 for PTV	100	20	
Pre-existing pulmonary diseases			
IP	7	1	0.196 (Chi-squared test)
COPD	35	8	
IP and COPD	3	1	
Neither	197	20	

RP: radiation pneumonitis, KL-6: Serum Krebs von den Lungen-6, PTV: planning target volume, IP: interstitial pneumonia, COPD: chronic obstructive pulmonary disease.

**Table 2:** Demographic distributions of the patients, whose pretreatment data of serum Krebs von den Lungen-6 (KL-6 database) was available, and significant differences between the training and test datasets.

	Training dataset	Test dataset	p-value (testing method)
Total number of cases	193	25	
RP grade			
Grade = 1	177	19	0.0275 (Chi-squared test)
Grade ≥ 2	16	6	
Pre-treatment KL-6 quantity (U/mL, min-max (median))	115–1853 (262)	134–678 (283)	0.745 (Mann-Whitney U-test)
Age (y, min-max (median))	51–91 (77)	54–90 (75)	0.902 (Mann-Whitney U-test)
Sex			0.268 (Chi-squared test)
Male	121	17	
Female	72	8	
Stage			0.220 (Chi-squared test)
T1N0M0 (stage IA)	144	22	
T2N0M0 (stage IB)	49	3	
Dose and fraction			
12 Gy × 4 Fr. (48 Gy)	188	21	9.50 × 10^−3^ (Chi-squared test)
13 Gy × 4 Fr. (52 Gy)	2	3	
6 Gy × 10 Fr. (60 Gy)	3	1	
Dose prescription method			
Isocenter	121	9	1.45 × 10^−2^ (Chi-squared test)
D95 for PTV	72	16	
Pre-existing pulmonary diseases			
IP	7	1	0.198 (Chi-squared test)
COPD	31	8	
IP and COPD	3	1	
Neither	152	15	

RP: radiation pneumonitis, KL-6: Serum Krebs von den Lungen-6, PTV: planning target volume, IP: interstitial pneumonia, COPD: chronic obstructive pulmonary disease.

## 3. Results

Table 1 shows the demographic distribution of the patients and the significant differences between the training and test datasets. In all, the data obtained from 242 cases (20 RP+ and 222 RP–) and 30 cases (8 RP+ and 22 RP–) were included for the training and testing of the RP prediction model, respectively. Since KL-6 information was available for 218 cases out of the 272 cases, the data (KL-6 datasets) were used to explore the synergistic combination of the BN with known predictive factors. Table 2 shows the demographic distributions of the KL-6 database and the significant differences between the training and test datasets.

**Fig 3:**
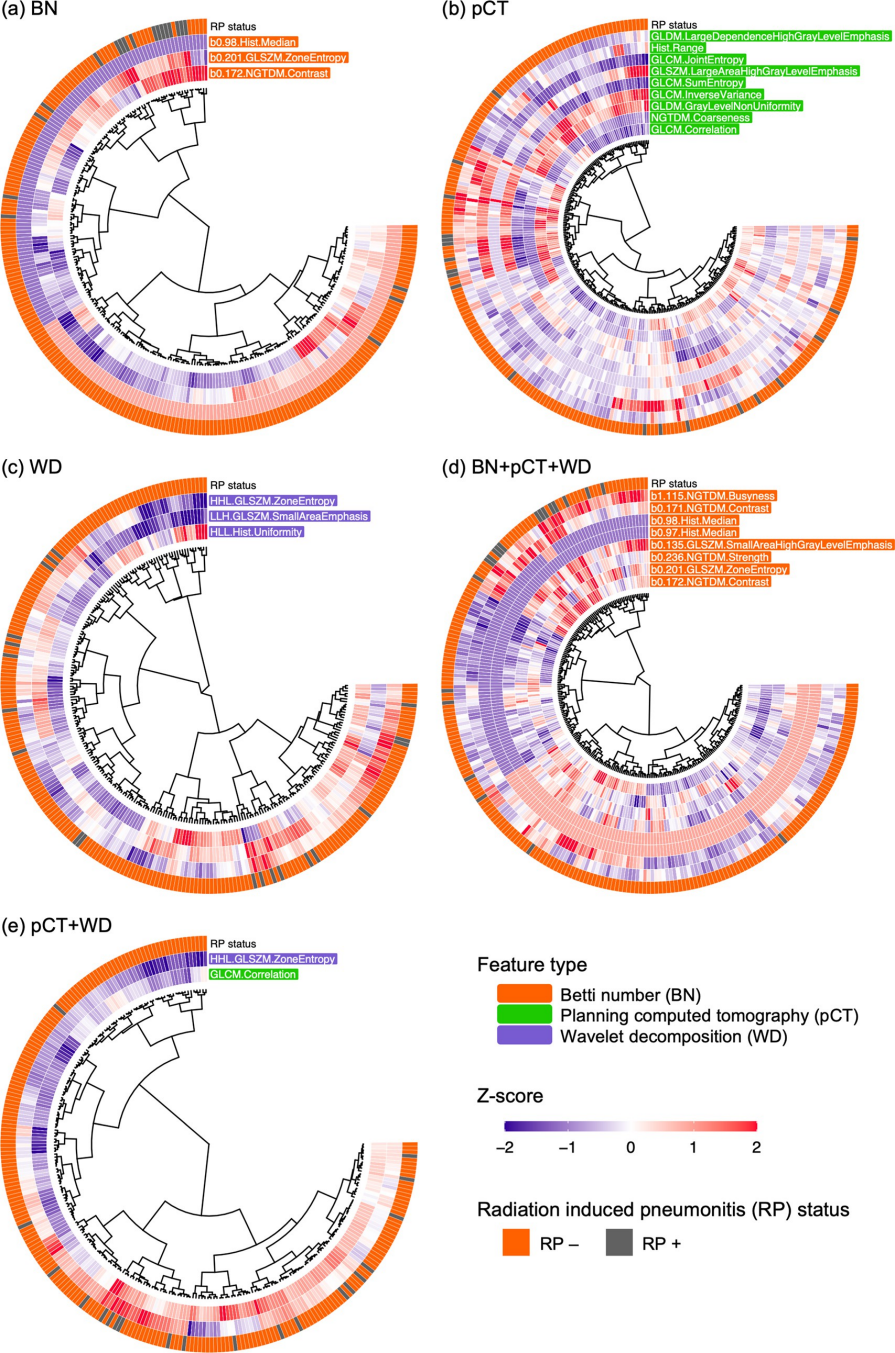
Selected features obtained from voting with LASSO logistic regression models based on (a) Betti number (BN), (b) planning computed tomography (pCT), (c) wavelet decomposition (WD) features, (d) a combination of all feature types (BN+pCT+WD) and a combination of two conventional feature types (pCT+WD).

Fig 3 depicts circular heatmaps representing the signatures constructed by voting using the LASSO-LR models for the BN, pCT, WD, BN+pCT+WD, and pCT+WD features. Descriptions of the features are shown in S2 Table. Three, nine, and three features were selected for the signatures of BN, pCT, and WD, respectively. The signature of BN+pCT+WD consisted of only BN features. For the conventional combination of pCT+WD, the signature was composed of two features, one from the pCT and the other from the WD.

The optimal parameter for the BN maps was a kernel size of 11 and a shifting pixel of 4. The optimal SVM parameters were a sigmoid kernel, gamma value of 0.4, and C margin of 0.1 (Table 3). For the BN+pCT+WD model, the optimal parameters were a sigmoid kernel, gamma value of 0.4, and C margin of 0.1. For the pCT+WD model, the optimal parameters were a linear kernel and a C margin of 0.1. For the pCT model, the optimal parameters were a sigmoid kernel, gamma value of 2.8, and C margin of 0.1. For the WD model, the optimal parameters were a sigmoid kernel, gamma value of 1.9, and C margin of 0.1.

**Table 3:** Summary of the areas under the receiver operating characteristics curves (AUCs) and robustness indices (RIs) obtained between the validation and test for the image signatures.

		Optimal SVM parameters			AUC (95% CI)		RI	Accuracy	Sensitivity	Specificity
	Number of features in the signature	Kernel	Gamma	C margin	Validation	Test				
BN	3	Sigmoid	0.4	0.1	0.873 (0.777–0.968)	0.852 (0.697–1.000)	1.691	0.757	0.756	0.757
BN+pCT+WD	8 (all BN)	Sigmoid	0.4	0.1	0.880 (0.810–0.950)	0.824 (0.651–0.997)	1.613	0.724	0.704	0.779
pCT+WD	2 (1 pCT, 1 WD)	Linear	-	0.1	0.775 (0.681–0.867)	0.790 (0.620–0.959)	1.541	0.681	0.664	0.728
pCT	9	Sigmoid	2.8	0.1	0.758 (0.656–0.860)	0.642 (0.417–0.867)	1.255	0.591	0.582	0.616
WD	3	Sigmoid	1.9	0.1	0.785 (0.700–0.870)	0.545 (0.296–0.795)	1.073	0.534	0.524	0.562

BN: Betti number, pCT: Planning computed tomography, WD: Wavelet-decomposition.

**Fig 4:**
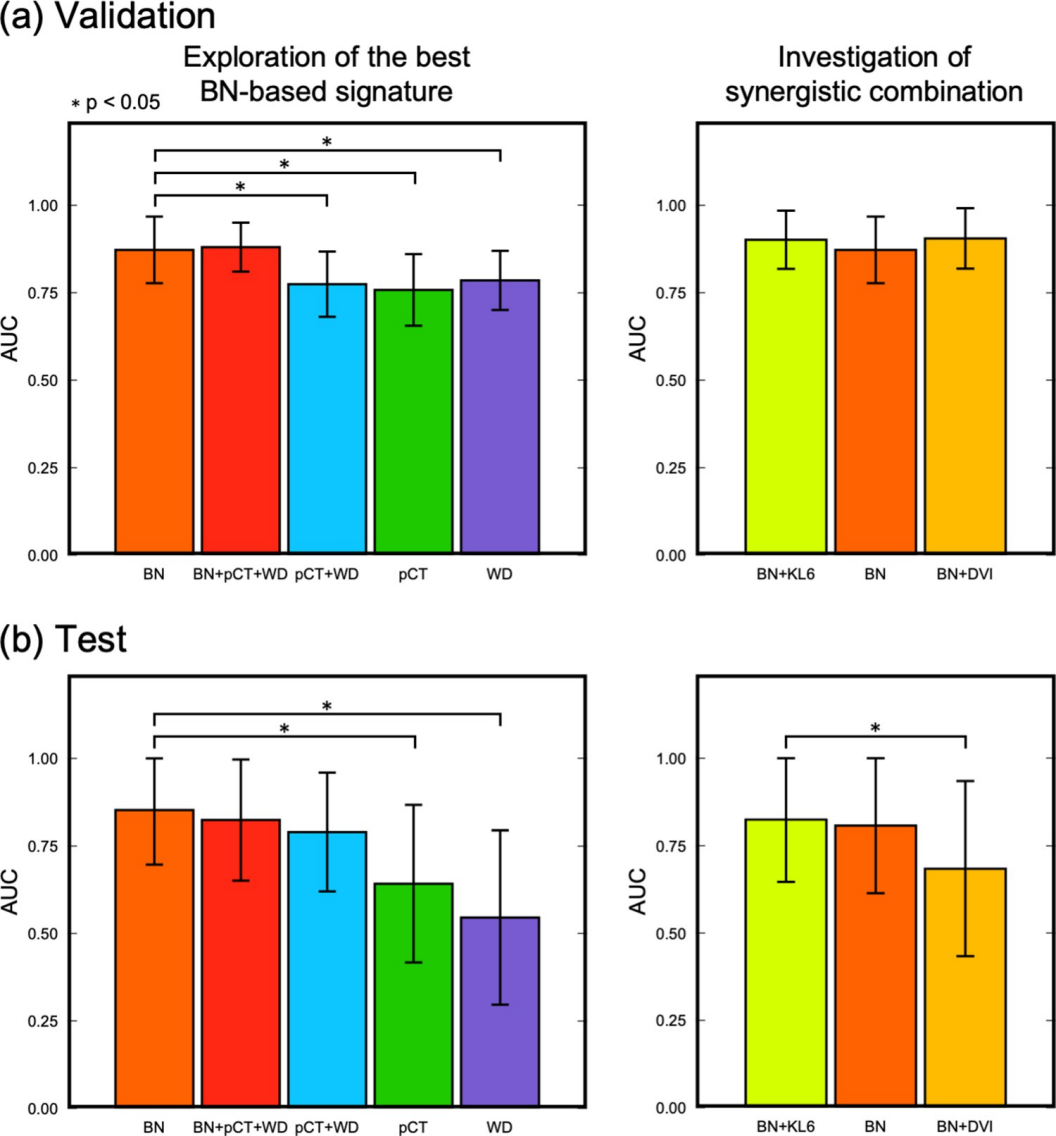
Comparison of the areas under the receiver operating characteristics curves (AUCs) among support vector machine models for (a) the validation and (b) the test with 95% confidence interval indicated by error bars. Bar graphs on the left side represent the results for the exploration of the best imaging biomarker, and those on the right side represent the results for the investigation of complementarity with known predictive factors.

Table 3 summarizes the AUCs, RIs, accuracy, sensitivity, and specificity of the RP+ prediction models based on the image signatures. The AUCs and the CIs that were obtained for the validation of the BN, BN+pCT+WD, pCT+WD, pCT and WD models were 0.873 (0.777–0.968), 0.880 (0.810–0.950), 0.775 (0.681–0.867), 0.758 (0.656–0.860) and 0.785 (0.700–0.870), respectively. In the validation, BN showed a significantly higher AUC than conventional models including pCT, WD, and pCT+WD (Fig 4). The AUCs and the CIs that were obtained for the test of the BN, BN+pCT+WD, pCT+WD, pCT and WD models were 0.852 (0.697–1.000), 0.824 (0.651–0.997), 0.790 (0.620–0.959), 0.642 (0.417–0.867) and 0.545 (0.296–0.795), respectively. In the test, the BN outperformed the conventional pCT and WD models, with statistically significant differences (Fig 4). The RIs obtained for the BN, BN+pCT+WD, pCT+WD, pCT, and WD models were 1.691, 1.613, 1.541 1.255, and 1.073, respectively. The accuracies (sensitivity and specificity) of the BN, BN+pCT+WD, pCT+WD, pCT, and WD models were found to be 0.757 (0.756, 0.757), 0.724 (0.704, 0.779), 0.681 (0.664, 0.728), 0.591 (0.582, 0.616), and 0.534 (0.524, 0.562), respectively.

Table 4 summarizes the results of the exploration of synergistic combination with KL-6. The AUCs and CIs that were obtained for the validation of the BN+KL-6, BN, and BN+DVI models were 0.901 (0.818–0.985), 0.873 (0.777–0.968), and 0.905 (0.819–0.992), respectively. The AUCs and CIs that were obtained for the test of the BN+KL-6, BN, and BN+DVI models were 0.825 (0.647–1.000), 0.807 (0.633–0.999), and 0.684 (0.434–0.587), respectively. BN+KL-6 showed higher AUCs than the BN model for both the validation and test. In contrast, although the BN+DVI model showed a higher AUC for the validation, it showed a significantly lower AUC than the BN model for the test (Fig 4). The RIs obtained for the BN+KL-6, BN, and BN+DVI models were 1.603, 1.576, and 1.302, respectively. The accuracies (sensitivity and specificity) of the BN+KL-6, BN, and BN+DVI models were found to be 0.724 (0.711, 0.765), 0.708 (0.699, 0.738), and 0.621 (0.618, 0.634), respectively.

**Table 4:** Summary of the areas under receiver operating characteristics curves (AUCs) and robustness indices (RIs) obtained between the validation and test for the Betti number (BN)-based signatures with serum Krebs von den Lungen-6 (KL-6) or dose volume indices (DVI).

		Optimal SVM parameters			AUC (95% CI)		RI	Accuracy	Sensitivity	Specificity
	Number of features in the signature	Kernel	Gamma	C margin	Validation	Test				
BN+KL6	4 (3BN, 1KL-6)	Sigmoid	0.1	0.4	0.901 (0.818–0.985)	0.825 (0.647–1.000)	1.603	0.724	0.711	0.765
BN	3	Sigmoid	0.4	0.1	0.873 (0.777–0.968)	0.807 (0.633–0.999)	1.576	0.708	0.699	0.738
BN+DVI	7 (3BN, 4DVI)	Sigmoid	0.1	0.4	0.905 (0.819–0.992)	0.684 (0.434–0.587)	1.302	0.621	0.618	0.634

## 4. Discussion

The results of the feature selection suggested the robustness and superiority of the BN features for RP+ prediction. The conventional pCT and WD features were not selected in the signature, although they were also included as candidates for the signature. The existence of conventional features affected the course of the feature selection based on voting, because the signature based on the BN+pCT+WD included nine features composed of six additional features apart from the three features selected in the BN-based signature. Nevertheless, the AUCs of the BN and BN+pCT+WD models did not show statistically significant differences in either the validation or test (Fig 4). Even though the constituents for the signature changed, the representative BN features were selected under consensus by voting with multiple LASSO-LR models, which consistently produced higher AUCs than the conventional models.

Table 5 compares the AUCs that were obtained in the present study to the AUCs obtained in previous studies. Our BN-based model outperformed the models reported in previous studies for RP prediction in SABR for lung cancer with respect to the test AUCs and RI [12, 13]. Although Hirose et al. reported a higher AUC for the validation than the BN model using the pCT+WD features, their model showed a lower AUC for the test [13]. In our study, we attempted to balance the AUCs for the pCT+WD model for the validation and test using RI. However, it showed a similar performance to the one reported by them. These results imply that the feasibility of conventional pCT and WD features is quite limited in RP prediction before SABR for lung cancer.

**Table 5:** Comparison of areas under the receiver operating characteristics curves (AUCs) obtained in the present study to the AUCs obtained in previous studies.

	Number of cases	Dataset	Feature type	AUC		RI
				Validation	Test	
The present study	218	Training 193	BN+KL-6	0.901	0.825	1.603
		Testing 25	BN	0.873	0.807	1.576
Hirose et al	275	Training 245	pCT+WD	0.871	0.756	1.459
		Testing 30				
Morgan et al	14	Not validated	pCT	0.750 (not validated)		–

BN: Betti number, KL-6: Serum Krebs von den Lungen-6, pCT: planning computed tomography, WD: wavelet decomposition.

The BN+KL-6 model improved the prediction, although it did not show a significant difference in comparison with the BN model. Although the use of KL-6 only cannot provide sufficient predictive performance, it could support RP+ prediction using the BN signature. All evaluation criteria including the RI, AUCs, accuracy, sensitivity, and specificity were improved in the BN+KL-6 model (Table 4). Although further investigations using larger datasets are needed, they may offer a reliable system for evaluating RP risk in lung cancer patients.

We also constructed the SVM model with BN-based signature, KL-6, and the status of prePDs. However, the model showed a similar predictive performance with the BN+KL-6 model. The AUC values for the validation and the test were 0.872 and 0.816, respectively. This might be because the model included the important phenotypes of the IP or COPD which is associated with the occurrence of the RP. The distributions of the outputs from the SVM model based on the BN+KL-6 showed a correlation with the status of pre-existing lung diseases (Table 6). Most of the IP-positive cases were predicted as RP+ by the model. On the other hand, two-third of all the COPD-positive cases were predicted as RP–. Those distributions of prePDs are similar to the observation reported in the previous studies; higher RP+ risk for the IP and lower RP+ risk for the COPD [14–18].

**Table 6:** Relationships between the predictions using the BN+KL-6 model and the pre-existing pulmonary diseases in the training dataset.

SVM prediction (BN+KL-6)	Status of pre-existing pulmonary diseases				Total
	IP	COPD	IP and COPD	Neither	
RP+	6	11	1	42	60
RP–	1	20	2	110	133

BN: Betti number, KL-6: Serum Krebs von den Lungen-6, IP: interstitial pneumonia, COPD: chronic obstructive pulmonary disease.

We investigated Spearman’s correlation coefficients between KL-6 and the three features in the BN-based signature and found no correlations among them (correlation coefficient <0.17). Therefore, the BN-based signature and KL-6 provide independent and useful measurements for RP+ prediction based on lung tissue conditions.

**Fig 5:**
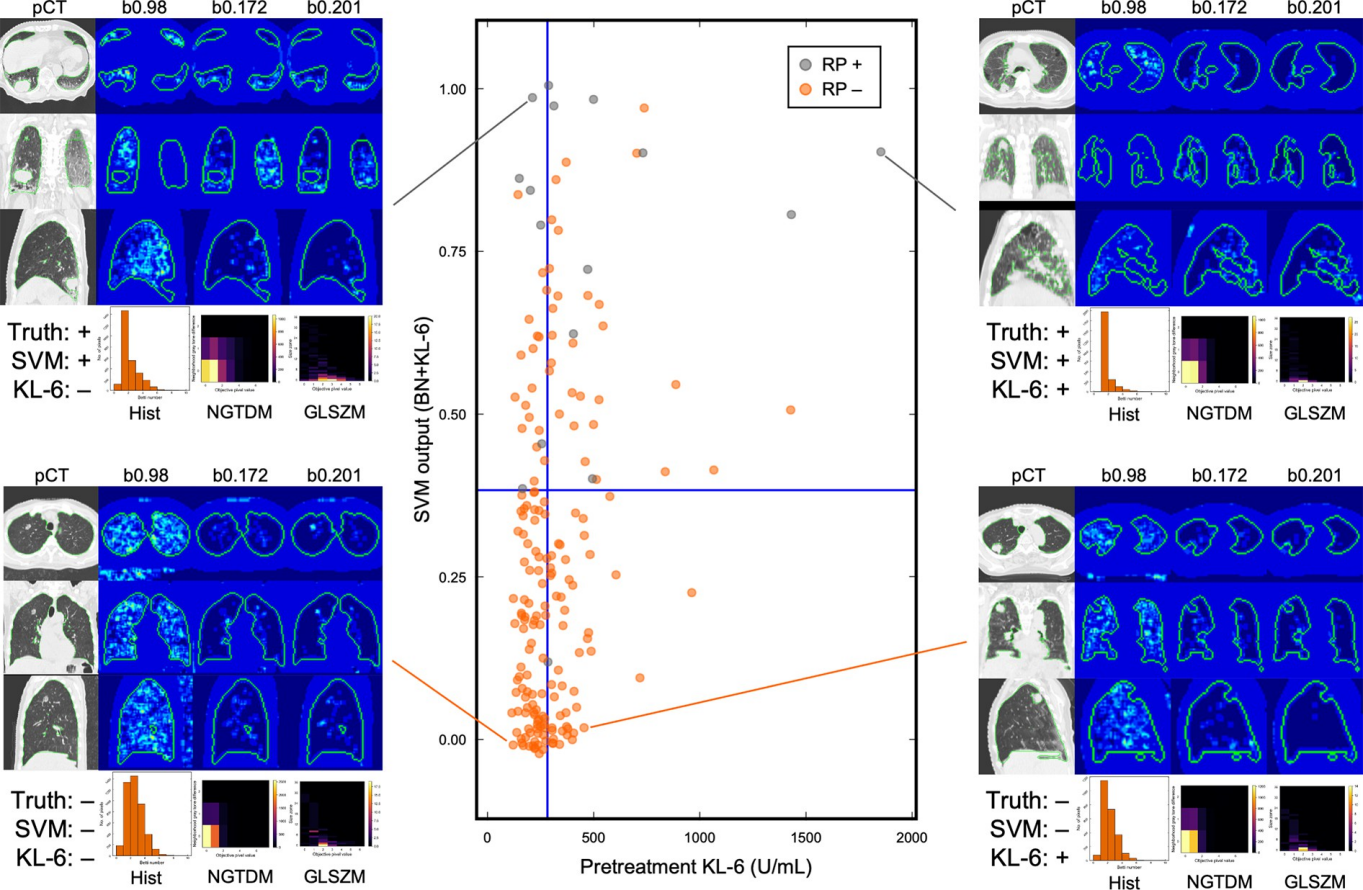
Distributions of the pretreatment serum Krebs von den Lungen-6 (KL-6) and support vector machine (SVM) outputs based on BN+KL-6. Representative features and Betti number (BN, b0) maps were shown for the cases where the SVM model could successfully predict the radiation pneumonitis (RP) status [positive (+) or negative (–)]. Labels of the BN maps (b0) are followed by the threshold value for obtaining the BN maps. Hist: histogram, NGTDM: neighborhood gray-tone difference matrix, GLSZM: gray-level size zone matrix.

The association between KL-6 levels and the severity of IP has been reported in previous studies [4, 34]. In this study, we found that KL-6 could detect patients with and without pre-existing IP in the training, as shown in the top right and bottom left examples in Fig 5, respectively. However, an increase in KL-6 does not always indicate the presence of IP. Yoshimatsu et al. reported that KL-6 levels could increase by the existence of lung cancer itself, with a significant association with tumor size [10]. Several cases showed high pre-treatment KL-6 levels, although they did not have pre-existing IP (the bottom right example in Fig 5). The sensitivity and specificity for the prediction of RP with KL-6 were found to be 0.582 and 0.625, respectively, using the optimal threshold value of 282.5 U/mL (vertical blue line in Fig 5), which is quite similar to that reported by Iwata et al. [9]. Contrastingly, the BN+KL-6 model could successfully predict the RP status in these patients. Furthermore, the model could predict the occurrence of RP in cases without IP or COPD (top left example in Fig 5). The sensitivity and specificity for the RP+ prediction with the BN+KL-6 model were found to be 0.746 and 0.938, respectively, using the optimal threshold value of 0.383 (horizontal blue line in Fig 5).

Variability of dose prescription methods and dose fractions may cause the lower performance of the BN+DVI model. In our database, which we have summarized in Tables 1 and 2, SABR plans were constructed using two dose prescription methods (isocenter or D95 for planning target volume) and three dose fractions [12 Gy×4 Fr. (48 Gy), 13 Gy×4 Fr. (52 Gy) or 6 Gy×10 Fr. Gy (60 Gy)]. Furthermore, there were statistically significant differences in these parameters between the training and test datasets. These differences affect the DVIs and can be attributed to poor prediction results with the BN+DVI model in the test.

The present study had four limitations. First, the BN model could not entirely evaluate the three-dimensional textures of the lung regions. Although we extended the range of the computation to the z-direction using the three cross-sections, it still utilizes two-dimensional computation algorithms for b0 and b1. Two-dimensional BNs cannot distinguish holes as intersections of the trachea from cavitation due to IP or COPD. Second, our model did not consider the impact of X-ray irradiation in SABR. As mentioned above, the DVIs did not improve the performance of RP+ prediction in our datasets. However, with respect to the RP+ prediction based on dosimetric information, Adachi et al. developed a model that implemented dosiomic features [35]. They showed a remarkable RP+ prediction performance that was much better than the DVI-based prediction. The dosiomic features could provide a critical dosimetric factor for RP+ prediction. Third, the present study could not clarify the feasibility of the model against other types of irradiation. Previous studies reported that RP could occur in several types of radiation treatments, such as conventional fractionated radiotherapy for lung cancer [36, 37] or head and neck regions [38]. Our model may reflect the vulnerability of lung regions to SABR for lung cancer. However, the generalizability of the findings obtained in the present study should be investigated in a wide variety of radiation treatments to provide comprehensive support for the risk assessment of RP in clinical practice. Last, we have not performed standardizations to normalize the impacts derived from the variations in the CT scanners. Since the BN features showed their robustness against the variations in the CT scanners in our previous study [25], we assumed that the BN features could mitigate the differences of the scanners in the training and testing datasets. However, it could show better prediction performance when the standardization was performed before the analysis. Haga et al reported that the classification performance of the histological subtypes of the early-stage lung cancer based on the imaging features extracted from CT images could be improved when standardization methods such as min-max normalization, z-score, or whitening from the principal component analysis were applied to the different (training and test) databases with different scanning conditions [39]. We intend to investigate the impact of standardization on the classification performance of the BN features in future work.

## 5. Conclusions

This study demonstrated the comprehensive performance of BNs for the prediction of potential RP+ patients before SABR for lung cancer. This prediction could be enhanced in combination with pretreatment with KL-6. We considered that the BN+KL-6 model was more feasible for the RP+ prediction because it showed the highest AUC value in the test of the exploration of synergistic combinations. These predictions could help radiation oncologists in decision making for dose reduction to avoid severe RP+ in personalized SABR or selection of alternative treatments to provide a better quality of life for each patient. Further studies with larger datasets and different radiation treatments to support the risk assessment of RP in clinical practice are needed.

## Supporting information

S1 Table Prediction performances of serum Krebs von den Lungen-6 (KL-6) and dose volume indices (DVI) of lung volume in the dataset used in the present study. The support vector machines were constructed for KL-6 and DVIs using the same ensemble strategy described in the main article.
(DOCX)

S2 Table Radiomic features with the feature types. (DOCX)

## References

[1] Li X, Niu C, Chen Q, Chen X. Comparison of efficacy of stereotactic body radiotherapy and thoracoscopic surgery in the treatment of early-stage non-small cell lung cancer. J BUON. 2020;25: 1497–1503. Available: https://www.ncbi.nlm.nih.gov/pubmed/32862596 32862596

[2] Tomita N, Okuda K, Osaga S, Miyakawa A, Nakanishi R, Shibamoto Y. Surgery versus stereotactic body radiotherapy for clinical stage I non-small-cell lung cancer: propensity score-matching analysis including the ratio of ground glass nodules. Clin Transl Oncol. 2020. doi: 10.1007/s12094-020-02459-8 32705493

[3] Yamashita H. Radiation pneumonitis after stereotactic radiation therapy for lung cancer. World J Radiol. 2014;6: 708. doi: 10.4329/wjr.v6.i9.708 25276313 PMC4176787

[4] Yoshitake T, Shioyama Y, Asai K, Nakamura K, Sasaki T, Ohga S, et al. Impact of interstitial changes on radiation pneumonitis after stereotactic body radiation therapy for lung cancer. Anticancer Res. 2015;35: 4909–4914. Available: https://www.ncbi.nlm.nih.gov/pubmed/26254387 26254387

[5] Yamashita H, Kobayashi-Shibata S, Terahara A, Okuma K, Haga A, Wakui R, et al. Prescreening based on the presence of CT-scan abnormalities and biomarkers (KL-6 and SP-D) may reduce severe radiation pneumonitis after stereotactic radiotherapy. Radiat Oncol. 2010;5: 1–9. doi: 10.1186/1748-717X-5-1 20459699 PMC2876174

[6] Hara R, Itami J, Komiyama T, Katoh D, Kondo T. Serum Levels of KL-6 for Predicting the Occurrence of Radiation Pneumonitis after Stereotactic Radiotherapy for Lung Tumors. Chest. 2004;125: 340–344. doi: 10.1378/chest.125.1.340 14718465

[7] Goto K, Kodama T, Sekine I, Kakinuma R, Kubota K, Hojo F, et al. Serum levels of KL-6 are useful biomarkers for severe radiation pneumonitis. Lung Cancer. 2001;34: 141–148. doi: 10.1016/s0169-5002(01)00215-x 11557124

[8] d’Alessandro M, Cameli P, Refini RM, Bergantini L, Alonzi V, Lanzarone N, et al. Serum KL-6 concentrations as a novel biomarker of severe COVID-19. J Med Virol. 2020;92: 2216–2220. doi: 10.1002/jmv.26087 32470148 PMC7283867

[9] Iwata H, Shibamoto Y, Baba F, Sugie C, Ogino H, Murata R, et al. Correlation between the serum KL-6 level and the grade of radiation pneumonitis after stereotactic body radiotherapy for stage i lung cancer or small lung metastasis. Radiother Oncol. 2011;101: 267–270. doi: 10.1016/j.radonc.2011.05.031 21640420

[10] Yoshimasu T, Oura S, Ota F, Hirai Y, Naito K, Nakamura R, et al. Journal of Pulmonary & Respiratory Serum KL-6 Levels in Patients with Lung Cancer. 2012;2. doi: 10.4172/2161-105X.10001

[11] d’Alessandro M, Bergantini L, Cameli P, Vietri L, Lanzarone N, Alonzi V, et al. Krebs von den Lungen-6 as a biomarker for disease severity assessment in interstitial lung disease: A comprehensive review. Biomark Med. 2020;14: 675–682. doi: 10.2217/bmm-2019-0215 32613855

[12] Moran A, Daly ME, Yip SSF, Yamamoto T. Radiomics-based Assessment of Radiation-induced Lung Injury After Stereotactic Body Radiotherapy. Clin Lung Cancer. 2017;18: e425–e431. doi: 10.1016/j.cllc.2017.05.014 28623121

[13] Hirose T-A, Arimura H, Ninomiya K, Yoshitake T, Fukunaga J-I, Shioyama Y. Radiomic prediction of radiation pneumonitis on pretreatment planning computed tomography images prior to lung cancer stereotactic body radiation therapy. Sci Rep. 2020;10: 20424. doi: 10.1038/s41598-020-77552-7 33235324 PMC7686358

[14] Ishihara T, Yamada K, Harada A, Yukiue H, Tanahashi M, Niwa H, et al. Stereotactic body radiotherapy for second primary lung cancer and intra-parenchymal lung metastasis in patients previously treated with surgery: evaluation of indications and predictors of decreased respiratory function. Acta Oncol. 2018;57: 1232–1239. doi: 10.1080/0284186X.2018.1468088 29722594

[15] Bajraszewski C, Manser R, Chu J, Cox RA, Tran P, Duffy M, et al. Adverse respiratory outcomes following conventional long-course radiotherapy for non-small-cell lung cancer in patients with pre-existing pulmonary fibrosis: A comparative retrospective study. J Med Imaging Radiat Oncol. 2020;64: 546–555. doi: 10.1111/1754-9485.13041 32386110

[16] Glick D, Lyen S, Kandel S, Shapera S, Le LW, Lindsay P, et al. Impact of Pretreatment Interstitial Lung Disease on Radiation Pneumonitis and Survival in Patients Treated With Lung Stereotactic Body Radiation Therapy (SBRT). Clin Lung Cancer. 2018;19: e219–e226. doi: 10.1016/j.cllc.2017.06.021 29066051

[17] Ishijima M, Nakayama H, Itonaga T, Tajima Y, Shiraishi S, Okubo M, et al. Patients with severe emphysema have a low risk of radiation pneumonitis following stereotactic body radiotherapy. Br J Radiol. 2015;88: 20140596. doi: 10.1259/bjr.20140596 25490255 PMC4614233

[18] Kimura T, Togami T, Takashima H, Nishiyama Y, Ohkawa M, Nagata Y. Radiation pneumonitis in patients with lung and mediastinal tumours: a retrospective study of risk factors focused on pulmonary emphysema. Br J Radiol. 2012;85: 135–141. doi: 10.1259/bjr/32629867 21385918 PMC3473945

[19] Larsen BT, Colby TV. Update for pathologists on idiopathic interstitial pneumonias. Arch Pathol Lab Med. 2012;136: 1234–1241. doi: 10.5858/arpa.2012-0225-RA 23020729

[20] Kadoch MA, Cham MD, Beasley MB, Ward TJ, Jacobi AH, Eber CD, et al. Idiopathic Interstitial Pneumonias: A Radiology-Pathology Correlation Based on the Revised 2013 American Thoracic Society-European Respiratory Society Classification System. Curr Probl Diagn Radiol. 2015;44: 15–25. doi: 10.1067/j.cpradiol.2014.07.005 25512168

[21] Brandsma CA, Van den Berge M, Hackett TL, Brusselle G, Timens W. Recent advances in chronic obstructive pulmonary disease pathogenesis: from disease mechanisms to precision medicine. J Pathol. 2020;250: 624–635. doi: 10.1002/path.5364 31691283 PMC7216938

[22] Hobbs S, Chung JH, Leb J, Kaproth-Joslin K, Lynch DA. Practical Imaging Interpretation in Patients Suspected of Having Idiopathic Pulmonary Fibrosis: Official Recommendations from the Radiology Working Group of the Pulmonary Fibrosis Foundation. Radiology: Cardiothoracic Imaging. 2021;3: e200279. doi: 10.1148/ryct.2021200279 33778653 PMC7977697

[23] Lynch DA, Austin JHM, Hogg JC, Grenier PA, Kauczor HU, Bankier AA, et al. CT-definable subtypes of chronic obstructive pulmonary disease: A statement of the fleischner society1. Radiology. 2015;277: 192–205. doi: 10.1148/radiol.2015141579 25961632 PMC4613878

[24] Ninomiya K, Arimura H. Homology-based approach for prognostic prediction of lung cancer using novel topologically invariant radiomic features. 2020. doi: 10.1117/12.2548918

[25] Ninomiya K, Arimura H, Chan WY, Tanaka K, Mizuno S, Muhammad Gowdh NF, et al. Robust radiogenomics approach to the identification of EGFR mutations among patients with NSCLC from three different countries using topologically invariant Betti numbers. Bianconi F, editor. PLoS One. 2021;16: e0244354. doi: 10.1371/journal.pone.0244354 33428651 PMC7799813

[26] Li J, Fong S, Mohammed S, Fiaidhi J. Improving the classification performance of biological imbalanced datasets by swarm optimization algorithms. J Supercomput. 2016. Available: https://link.springer.com/article/10.1007/s11227-015-1541-6

[27] Kumar P, Bhatnagar R, Gaur K, Bhatnagar A. Classification of Imbalanced Data:Review of Methods and Applications. IOP Conf Ser: Mater Sci Eng. 2021;1099: 012077. doi: 10.1088/1757-899X/1099/1/012077

[28] Branco P, Torgo L, Ribeiro R. A Survey of Predictive Modelling under Imbalanced Distributions. arXiv [cs.LG]. 2015. Available: http://arxiv.org/abs/1505.01658

[29] Sun Y, Wong AKC, Kamel MS. Classification of imbalanced data: A review. Int J Pattern Recognit Artif Intell. 2009;23: 687–719. doi: 10.1142/s0218001409007326

[30] Schiller TW, Chen Y, El Naqa I, Deasy JO. Modeling radiation-induced lung injury risk with an ensemble of support vector machines. Neurocomputing. 2010;73: 1861–1867. doi: 10.1016/j.neucom.2009.09.023

[31] Friedman J, Hastie T, Tibshirani R. Regularization Paths for Generalized Linear Models via Coordinate Descent. J Stat Softw. 2010;33: 1–22. doi: 10.1016/j.expneurol.2008.01.011 20808728 PMC2929880

[32] Palma DA, Senan S, Tsujino K, Barriger RB, Rengan R, Moreno M, et al. Predicting radiation pneumonitis after chemoradiation therapy for lung cancer: An international individual patient data meta-analysis. Int J Radiat Oncol Biol Phys. 2013;85: 444–450. doi: 10.1016/j.ijrobp.2012.04.043 22682812 PMC3448004

[33] Ryckman JM, Baine M, Carmicheal J, Osayande F, Sleightholm R, Samson K, et al. Correlation of dosimetric factors with the development of symptomatic radiation pneumonitis in stereotactic body radiotherapy. Radiat Oncol. 2020;15: 33. doi: 10.1186/s13014-020-1479-6 32054487 PMC7020355

[34] Qin H, Xu XP, Zou J, Zhao XJ, Wu HW, Zha QF, et al. Krebs von den Lungen-6 associated with chest high-resolution CT score in evaluation severity of patients with interstitial lung disease. Pulmonology. 2019;25: 143–148. doi: 10.1016/j.pulmoe.2018.05.008 30007895

[35] Adachi T, Nakamura M. Multi-institutional dose-segmented dosiomic analysis for predicting radiation pneumonitis after lung stereotactic body radiation therapy. 2021;48: 1781–1791. doi: 10.1002/mp.14769 33576510

[36] Cui S, Luo Y, Tseng HH, Ten Haken RK, El Naqa I. Combining handcrafted features with latent variables in machine learning for prediction of radiation-induced lung damage. Med Phys. 2019;46: 2497–2511. doi: 10.1002/mp.13497 30891794 PMC6510637

[37] Luna JM, Chao HH, Diffenderfer ES, Valdes G, Chinniah C, Ma G, et al. Predicting radiation pneumonitis in locally advanced stage II–III non-small cell lung cancer using machine learning. Radiother Oncol. 2019;133: 106–112. doi: 10.1016/j.radonc.2019.01.003 30935565

[38] Cunliffe A, Armato SG, Castillo R, Pham N, Guerrero T, Al-Hallaq HA. Lung texture in serial thoracic computed tomography scans: Correlation of radiomics-based features with radiation therapy dose and radiation pneumonitis development. Int J Radiat Oncol Biol Phys. 2015;91: 1048–1056. doi: 10.1016/j.ijrobp.2014.11.030 25670540 PMC4383676

[39] Haga A, Takahashi W, Aoki S, Nawa K, Yamashita H, Abe O, et al. Standardization of imaging features for radiomics analysis. J Med Invest. 2019;66: 35–37. doi: 10.2152/jmi.66.35 31064950

