# Supplementary material for: Synergistic combination of a topologically invariant imaging signature and a biomarker for the accurate prediction of symptomatic radiation pneumonitis before stereotactic ablative radiotherapy for lung cancer: A retrospective analysis
Source: PLoS One. 2022 Jan 31;17(1):e0263292. doi: 10.1371/journal.pone.0263292 (PMC8803154; doi:10.1371/journal.pone.0263292)
Supplement: S1 Table — The support vector machines were constructed for KL-6 and DVIs using the same ensemble strategy described in the main article. (DOCX) [file pone.0263292.s001.docx]

**S1 Table. Prediction performances of serum Krebs von den Lungen-6 (KL-6) and dose volume indices (DVI) of﻿ lung volume in the dataset used in the present study.** The support vector machines were constructed for KL-6 and DVIs using the same ensemble strategy described in the main article.

|  | AUC | | Robustness index |
| --- | --- | --- | --- |
|  | Validation | Test |  |
| KL-6 | 0.626 | 0.333 | 0.742 |
| DVI | 0.610 | 0.246 | 0.627 |

AUC: area under the receiver operating characteristics curves
