# Supplementary material for: Synergistic combination of a topologically invariant imaging signature and a biomarker for the accurate prediction of symptomatic radiation pneumonitis before stereotactic ablative radiotherapy for lung cancer: A retrospective analysis
Source: PLoS One. 2022 Jan 31;17(1):e0263292. doi: 10.1371/journal.pone.0263292 (PMC8803154; doi:10.1371/journal.pone.0263292)
Supplement: S2 Table — (DOCX) [file pone.0263292.s002.docx]

| **S2 Table. Radiomic features with the feature types.** | | | | | |
| --- | --- | --- | --- | --- | --- |
| Histogram-based features (n = 18) | Texture features (n = 75) | | | | |
|  | GLCM (n = 24) | GLSZM (n = 16) | GLRLM (n = 16) | NGTDM (n = 5) | GLDM (n =14) |
| 1. Energy 2. Total Energy 3. Entropy 4. Minimum 5. 10^th^ percentile 6. 90^th^ percentile 7. Maximum 8. Mean 9. Median 10. Interquartile range 11. Range 12. Mean Absolute Deviation (MAD) 13. Robust Mean Absolute Deviation (rMAD) 14. Root Mean Squared (RMS) 15. Skewness 16. Kurtosis 17. Variance 18. Uniformity | 1. Autocorrelation 2. Joint Average 3. Cluster Prominence 4. Cluster Shade 5. Cluster Tendency 6. Contrast 7. Correlation 8. Difference Average 9. Difference Entropy 10. Difference Variance 11. Joint Energy 12. Joint Entropy 13. Informational Measure of Correlation (IMC) 1 14. Informational Measure of Correlation (IMC) 2 15. Inverse Difference Moment (IDM) 16. Maximal Correlation Coefficient (MCC) 17. Inverse Difference Moment Normalized (IDMN) 18. Inverse Difference (ID) 19. Inverse Difference Normalized (IDN) 20. Inverse Variance 21. Maximum Probability 22. Sum Average 23. Sum Entropy 24. Sum of Squares | 1. Small Area Emphasis (SAE) 2. Large Area Emphasis (LAE) 3. Gray Level Non-Uniformity (GLN) 4. Gray Level Non-Uniformity Normalized (GLNN) 5. Size-Zone Non-Uniformity (SZN) 6. Size-Zone Non-Uniformity Normalized (SZNN) 7. Zone Percentage (ZP) 8. Gray Level Variance (GLV) 9. Zone Variance (ZV) 10. Zone Entropy (ZE) 11. Low Gray Level Zone Emphasis (LGLZE) 12. High Gray Level Zone Emphasis (HGLZE) 13. Small Area Low Gray Level Emphasis (SALGLE) 14. Small Area High Gray Level Emphasis (SAHGLE) 15. Large Area Low Gray Level Emphasis (LALGLE) 16. Large Area High Gray Level Emphasis (LAHGLE) | 1. Short Run Emphasis (SRE) 2. Long Run Emphasis (LRE) 3. Gray Level Non-Uniformity (GLN) 4. Gray Level Non-Uniformity Normalized (GLNN) 5. Run Length Non-Uniformity (RLN) 6. Run Length Non-Uniformity Normalized (RLNN) 7. Run Percentage (RP) 8. Gray Level Variance (GLV) 9. Run Variance (RV) 10. Run Entropy (RE) 11. Low Gray Level Run Emphasis (LGLRE) 12. High Gray Level Run Emphasis (HGLRE) 13. Short Run Low Gray Level Emphasis (SRLGLE) 14. Short Run High Gray Level Emphasis (SRHGLE) 15. Long Run Low Gray Level Emphasis (LRLGLE) 16. Long Run High Gray Level Emphasis (LRHGLE) | 1. Coarseness 2. Contrast 3. Busyness 4. Complexity 5. Strength | 1. Small Dependence Emphasis (SDE) 2. Large Dependence Emphasis (LDE) 3. Gray Level Non-Uniformity (GLN) 4. Dependence Non-Uniformity (DN) 5. Dependence Non-Uniformity Normalized (DNN) 6. Gray Level Variance (GLV) 7. Dependence Variance (DV) 8. Dependence Entropy (DE) 9. Low Gray Level Emphasis (LGLE) 10. High Gray Level Emphasis (HGLE) 11. Small Dependence Low Gray Level Emphasis (SDLGLE) 12. Small Dependence High Gray Level Emphasis (SDHGLE) 13. Large Dependence Low Gray Level Emphasis (LDLGLE) 14. Large Dependence High Gray Level Emphasis (LDHGLE) |

GLCM: gray-level co-occurrence matrix, GLSZM: gray-level size zone matrix, GLRLM: gray-level run-length matrix, NGTDM: neighborhood gray-tone difference matrix, GLDM, gray-level dependence matrix.

[Reference]

Pyradiomics, radiomic features; available at <https://pyradiomics.readthedocs.io/en/latest/features.html>, accessed May 10^th^, 2021.
